# The Role of Bone Morphogenetic Protein 7 (BMP-7) in Inflammation in Heart Diseases

**DOI:** 10.3390/cells9020280

**Published:** 2020-01-23

**Authors:** Chandrakala Aluganti Narasimhulu, Dinender K Singla

**Affiliations:** Division of Metabolic and Cardiovascular Sciences, Burnett School of Biomedical Sciences, College of Medicine, University of Central Florida, Orlando, FL 32816, USA; Chandrakala.AlugantiNarasimhulu@ucf.edu

**Keywords:** atherosclerosis, myocardial infarction, diabetic cardiomyopathy, inflammation

## Abstract

Bone morphogenetic protein-7 is (BMP-7) is a potent anti-inflammatory growth factor belonging to the Transforming Growth Factor Beta (TGF-β) superfamily. It plays an important role in various biological processes, including embryogenesis, hematopoiesis, neurogenesis and skeletal morphogenesis. BMP-7 stimulates the target cells by binding to specific membrane-bound receptor BMPR 2 and transduces signals through mothers against decapentaplegic (Smads) and mitogen activated protein kinase (MAPK) pathways. To date, rhBMP-7 has been used clinically to induce the differentiation of mesenchymal stem cells bordering the bone fracture site into chondrocytes, osteoclasts, the formation of new bone via calcium deposition and to stimulate the repair of bone fracture. However, its use in cardiovascular diseases, such as atherosclerosis, myocardial infarction, and diabetic cardiomyopathy is currently being explored. More importantly, these cardiovascular diseases are associated with inflammation and infiltrated monocytes where BMP-7 has been demonstrated to be a key player in the differentiation of pro-inflammatory monocytes, or M1 macrophages, into anti-inflammatory M2 macrophages, which reduces developed cardiac dysfunction. Therefore, this review focuses on the molecular mechanisms of BMP-7 treatment in cardiovascular disease and its role as an anti-fibrotic, anti-apoptotic and anti-inflammatory growth factor, which emphasizes its potential therapeutic significance in heart diseases.

## 1. Introduction

In 1970, a physician named Marshall Urist coined the term bone morphogenetic protein (BMP) after demonstrating that these proteins play an important role in osteogenesis and bone formation. Thereafter, more than 20 BMPs have been identified and subdivided into the following four groups; (i) BMP-2/4, (ii) BMP-5/6/7/8a/8b, (iii) BMP-9/10, and (iv) BMP-12/13/14 based on their function and amino acid sequence similarity [1,2,3,4]. BMP signaling plays a crucial role in several developmental pathways. BMPs regulate erythropoiesis and neurogenesis during embryonic development by interacting with the BMP receptors (BMPR) I and II [5,6]. Accordingly, their function in embryogenesis has been extensively studied in several model organisms including frogs, mice, and zebrafish. After birth, they maintain bone mass by inducing the differentiation of mesenchymal stem cells (MSCs) into osteoblasts and regulating their differentiation potential [7,8,9,10,11,12]. Specifically, BMP-2/4/6/7/9/12/13 have the ability to induce MSC differentiation, whereas BMP-3 plays a role in inducing MSC proliferation [7,8,9,10,11,12,13]. In addition to MSCs, existing studies reveal that adipocyte, fibroblast, myoblast and neural cell differentiation and proliferation is also regulated by BMPs [14,15,16,17,18].

Evidence suggests that BMP-2, 4 and 10 deletion is embryonically lethal [19,20,21] whereas loss of BMP-7/11 leads to death immediately after birth [22,23]. Moreover, deletion of BMP receptors [24,25,26] and downstream transducers (Smad-1/4/5/7) are also embryonically lethal [27,28,29,30]. BMP-4 insufficiency prompted an imbalance in the hematopoietic stem cell (HSC) proliferation and differentiation, whereas the lack of BMP-4 has disrupted gastrulation and subsequent formation of the mesoderm, obstructing the generation of major tissues such as cardiac, skeletal, and vascular muscle cells that resulted in animal lethality [31]. BMP-2/10 play a key role in myocardial patterning, chamber formation and maturation [21,32,33]. The diverse biological activities of BMPs [3,25,26] along with their receptors are highlighted in Table 1 [34,35,36], and it is clear that BMP deficiency can result in numerous human pathophysiological diseases and death.

In addition to BMP receptors, BMP-7 also exerts its biological effects through the type 1 and type 2 receptors of activin [35,36]. It has been reported that BMP-7 deletion leads to death and its deficiency induces different diseases such as osteoporosis. Therefore, BMP-7 was used for the treatment of osteoporosis [37,38,39], a widespread condition affecting several millions of people worldwide. This disease is characterized by the loss of bone mineral density, resulting in an increased susceptibility to osteoporosis induced bone fracture [40,41,42]. However, further studies are required to understand the role of BMP-7 in tissue-specific disease development and therapeutic applications. In recent years, the use of BMP-7 has been extended to several other inflammatory diseases, including cardiovascular diseases (CVD) and cellular plasticity to neurological disorders. Therefore, the focus of this review article was to provide an overall structure of BMP-7, mechanistic pathways and its potential therapeutic significance in CVD.

## 2. Structure of BMP-7

**Figure 1:**
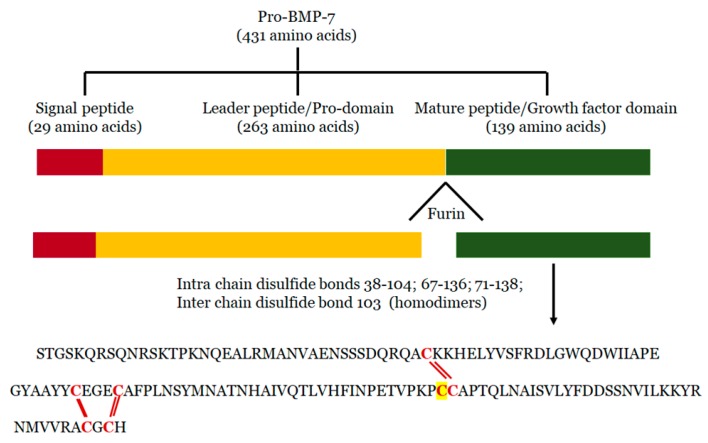
BMP-7 Structure: During processing, Pro-BMP-7 hydrolyzation by Furin on its carboxy terminal and converts into BMP-7 with three intra-chain disulfide bond forming cysteine residues and one inter-chain disulfide bond forming cysteine residue (highlighted).

BMP-7 is expressed by several tissues, including, sensory organs (eye and skin), major end organs (heart, lung, liver, pancreas, kidney, and brain), lymphoid organs (bone marrow, thymus and lymph nodes), the reproductive system (testis, ovary, uterus and placenta), exocrine glands (prostate and mammary gland), and organ protectors (muscle and bone) [22,43,44,45,46,47,48,49]. It is synthesized in the cells as pro-protein form of 431 amino acid residues, including *N*-terminal signal peptide of 29 amino acid residues, a pro-peptide of 263 amino acids, and a mature peptide of 139 amino acid residues [50] (Figure 1). During processing, pro-BMP-7 is hydrolyzed in the cell by furin-like proteinase on its carboxy terminal, where it is converted into mature BMP-7 of 139 amino acid residues and secreted into the extracellular matrix [51]. BMP-7 is approximately a 35 kDa glycoprotein with three *N*-glycosylation sites and seven cysteine residues involved in three intramolecular disulfide bonds Cys38-104, Cys67-136 and Cys71-138 [52]. More importantly the intermolecular disulfide bond formed via the 103rd cysteine form dimers in two mature BMP-7 monomers with enhanced biological activity. BMP-7 has the ability to form homodimers as well as heterodimers to induce bone formation. It has been reported that BMP-7 can form heterogenous dimers with other BMPs, specifically, BMP-2 and BMP-4 [53,54,55]. However, heterodimers are more potent than homodimers in osteogenic differentiation assays [56,57,58]. Moreover, it has been demonstrated that the biological activity of these heterogenous dimers is almost 20 times higher than that of homodimers [39,58,59]. These heterodimers also showed enhanced activity in embryonic assays of Xenopus and Zebrafish [60,61]. According to these studies, co-injection of RNA encoding BMP-7 with BMP-2 or BMP-4 into embryonic blastomere enhanced embryo ventralization and patterning compared with individual injection. Additionally, combined injection of purified recombinant proteins of BMP4/7 or BMP2/7 increased BMP signaling (SMAD pathway) in Xenopus and Zebrafish, whereas varied concentrations of individual injections of homodimers did not have that level of BMP signaling alterations, suggesting that heterodimes are more potent in BMP cell signaling [55,61,62].

Recently, to evaluate the heterodimer presence in vivo, Kim et al. generated knock in mice carrying a mutation (Bmp7R-GFlag) that prevents proteolytic activation of the dimerized BMP-7 precursor protein [63]. This mutation abolishes the ability of BMP-7 homo and heterodimer formation. Further, the presence of endogenous BMP4/7 heterodimer was confirmed with coimmunoprecipitation assays. These studies suggested that BMP-7 predominantly forms heterodimers with BMP-2 or BMP-4 and plays a major role during mammalian development.

BMP-7 is a pleiotropic growth factor and plays a crucial role in the development of various tissues and organs as represented in Table 1. It maintains multiple physiological processes such as bone development, fracture healing, and differentiation of brown adipose tissue in the body. Reduction in BMP-7 expression is associated with various diseases including osteoporosis, CVD and diabetes. In 1980, the recombinant human BMP-7 (rhBMP-7) expressed in Chinese hamster ovary cells was approved to use as a therapeutic agent in the repair of bone fractures and has been successfully implemented in clinical trials [64,65,66,67]. Moreover, BMP-7containing osteogenic implants have been used widely for the treatment of long bone non-unions, spinal fusions, and acute fractures [68]. In addition, earlier reports from our laboratory have demonstrated the potential protective role of BMP-7 in inhibiting plaque formation, monocyte infiltration and in the inhibition of pro-inflammatory cytokine secretion [69,70]. Further, we also observed reduced circulatory BMP-7 levels as atherosclerosis progressed and that the exogenous supplementation of BMP-7 significantly attenuated disease progression [71]. Recent studies revealed that BMP-7 not only reduces body fat, but also strengthens insulin signaling, further improves glucose uptake and insulin resistance [72]. Considering the beneficial effects of BMP-7 in metabolism, this review focuses on the molecular aspects of BMP-7 and its regulation in inflammation in CVD. The current literature has suggested the therapeutic efficacy of BMP-7 mediated through canonical and non-canonical mechanistic pathways in various animal disease models of CVD, diabetes and obesity [65,66].

## 3. Mechanisms of BMP-7

**Figure 2:**
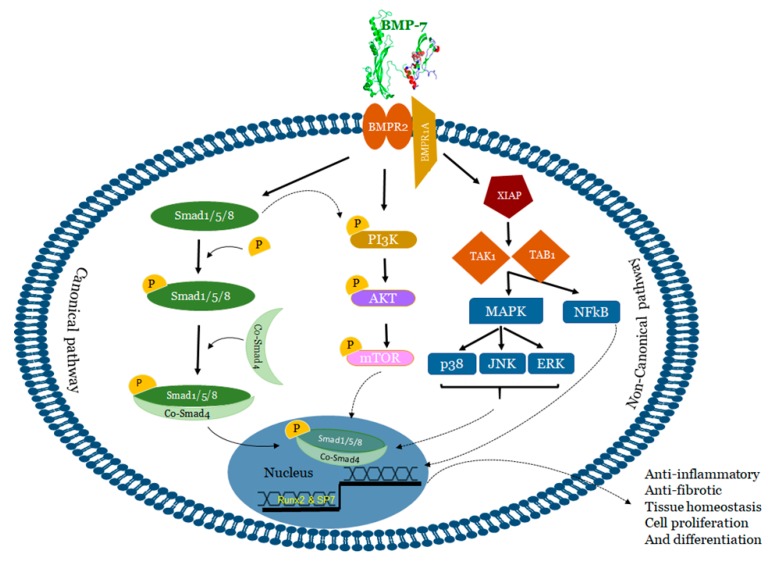
BMP signaling pathways. BMP-7 transduces signals in target cells by binding to a specific membrane bound receptor BMPR2 and phosphorylates BMPR1, which activates both canonical and non-canonical pathways. In the canonical pathway, activated BMPR2 leads to phosphorylation of Smad-1/5/8 which complexes with Smad-4 and translocate the signal. In the non-canonical pathway, p38 MAPK, JNK, ERK and NFKB were activated via the activation of XIAP, TAK1 and TAB1 whereas PI3K, Akt were activated by both BMPR2 and Smad-1/5/8. Altogether, this influences the different transcription factors and regulates the gene expression. BMP: Bone morphogenetic protein; BMPR: Bone morphogenetic protein receptor; XIAP: X-linked inhibitor of apoptosis protein; TAK1:TGF-beta activated kinase 1; TAB1: TAK1 binding protein; Runx2: Run-related transcription factor 2; MAPK: Mitogen-activated protein kinase; JNK: c-Jun-N terminal Kinase; ERK: Extracellular signal-regulated kinase; PI3K: Phosphotidylinositol 3 kinase; Akt: RAC-alpha serine/threonine-protein kinase; mTOR: mammalian target of rapamycin.

BMP-7 binds to bone morphogenetic protein receptor 2 (BMPR2) on the surface of cells and activates two major signaling pathways: 1) Canonical/Smad dependent and 2) Non-canonical/Smad independent pathway [65,66] (Figure 2).

In the canonical or Smad dependent pathway (Figure 2), BMP-7 activates regulatory Smads (Smad-1, 5, and 8) for subsequent phosphorylation in the cytoplasm. Thereafter, phosphorylated regulatory Smad proteins form a complex with the co-stimulatory molecule Smad-4. This complex is then transduced to the nuclei to recruit cofactors and Run-related transcription factor 2 (Runx2) to regulate osteogenic gene expression and consequently influences osteoblast differentiation [65,73,74]. Mesenchymal stem cell differentiation into osteoblasts is a pre-requisite for embryonic skeletal formation, homeostatic skeletal remodeling and bone fracture repair. BMP-7 plays a major role in upregulating the transcription factor osterix (Osx) or SP7 which has the ability to stimulate differentiation of osteoblasts both in vitro and in vivo [65,75,76]. These studies suggested the involvement of canonical cell signaling pathway in osteoblast differentiation and embryo skeletal formation induced by BMP-7 [76,77,78,79,80,81,82]. BMP-7 induced activation of Smad-1/5 leads to the activation of osterix resulting in increased osteogenic markers alkaline phosphatase (ALP) activity and mineralization [83]. Lavery et al. demonstrated the BMP-7 mediated osteoblastic differentiation of primary human mesenchymal stem cells with strongly enriched established osteogenic marker genes including osteocalcin (OCN), osteopontin (OPN) and ALP along with several other osteogenic markers of unknown function [84]. It has been reported that BMP-7 differentiates murine C2C12 myoblasts into osteoblasts by suppressing myoblast determination protein 1 (MyoD) expression, and enhancing the ALP activity and the osteogenic specific gene expressions ALP, Runx2, and OCN via P38 mitogen-activated protein kinase (MAPK) dependent Smad-1/5/8 signaling pathways [85]. Alongside, a recent study from our laboratory demonstrated monocyte differentiation into anti-inflammatory M2 macrophages through the Smad-1/5/8 pathway [67].

On the other hand, in the non-canonical pathway (Figure 2), BMP-7 transduces the signal to the MAPK signaling via c-Jun-*N* terminal kinase (JNK)1/2/3, extracellular signal-regulated kinase (ERK)1/2, nuclear factor kappa-light-chain-enhancer of activated B (NFκB), and p38 to regulate different target gene expressions [86,87]. Activated BMPR1A receptor complex initiates these pathways through a series of protein interactions including bone morphogenetic protein receptor associated molecule 1 (BRAM1) or X-linked inhibitor of apoptosis protein (XIAP), and downstream signaling molecules TGF-beta activated kinase 1 (TAK1) and TAK1 binding protein (TAB1) [88]. TAK1 and TAB1 binding activates downstream NFkB, p38, and JNK pathways that induces cell death and differentiation [86,87,88,89]. In addition, BMP-7 activates ERK, Phosphoinositol 3-kinase (PI3K), Protein Kinase (PK) C, and D which play a role in cell survival, apoptosis, migration and differentiation [90,91,92].

Hu et al. showed that BMP-7 stimulates renal epithelial cell morphogenesis via p38 MAPK and that its action is counteracted by Smad-1. Further, these studies also revealed that responses to low doses of BMP-7 lead to increased cell proliferation, which are regulated by the p38 MAPK pathway while responses to high doses of BMP-7 suppress cell proliferation, and are controlled by the Smad pathway. In addition, suppression of the p38 MAPK activity by high doses of BMP-7 might integrate the dose-dependent cellular response to BMP-7 [93].

BMP-7 promotes proliferation of nephron progenitor cells through TAK1-mediated JNK activation as well as further activation of transcription factor Jun and activating transcription factor 2 (ATF2) [94]. BMP-7 also plays a major role in the induction of tissue factor in human mononuclear cells (MNCs) through NF-KB activity, leading to increased F3 (tissue factor gene) transcription [95] and resulting in an increased procoagulant activity.

Additionally, it has been noticed that BMP-7 binding to its receptor BMPR-II can also activate the Smad dependent and independent PI3K pathways. In this process, activation of PI3K subunit p85 occurs either by Smad-1/5/8 or BMP-7 binding to BMPR II and its subsequent phosphorylation leads to down-stream phosphorylation of phosphotidylinositol biphosphate (PIP2) to phosphatidylinositol triphosphate (PIP3) [96,97] which, in turn, leads to the phosphorylation of RAC-alpha serine/threonine-protein kinase (Akt) and downstream activation of mammalian target of rapamycin (mTOR) [98]. In immune regulation, the PI3K pathway plays an important role in maintaining the anti-inflammatory environment [97]. Furthermore, studies from our laboratory demonstrated that the Smad-PI3K-Akt-mTOR pathway specifically inhibits pro-inflammatory cytokine secretion (TNF-α, IL-6 and MCP-1), enhances anti-inflammatory cytokines (IL-10 and IL-1ra) and plays a key role in M2 macrophage polarization [67,70].

## 4. Inhibitors of BMP-7

Several extra- and intra-cellular regulators, which play a major role in BMP signaling pathways via binding receptors and blocking pathways have been identified. Almost 15 BMP antagonists have been identified and classified into four major groups based on the size and cysteine knot as represented in Table 2 [3,99,100,101,102,103,104]. Similarly, intracellular BMP signaling is inhibited by micro-RNAs, I-Smads (Smad-6 and 7) and phosphatases (PP1 and PP2A) which play a role in dephosphorylation of both phosphorylated R-Smads and type I receptors [105,106,107,108]. Noggin, chordin and follistatin have been considered as major antagonists for BMP-7 [99,109,110,111]. Noggin blocks the effects of BMP-7 in osteoblast differentiation and inhibits membrane ossification and further limb development [99,109]. Similarly, Chordin stops binding of BMP-7 to the receptor and further the phosphorylation of down-stream proteins, resulting in inhibition of several biological functions [110]. Follistatin inhibits the binding of the BMP-7 to BMPR2 and prevents the activation of the Smad-1/5/8 pathway [111].

BMP antagonists also play a crucial role in embryonic development. To elaborate, embryogenesis is mediated by the activity of extracellular proteins such as chordin, noggin, cerberus, and dan family protein gremlin2. Amongst these antagonists, gremlin2 acts as the strongest BMP ligand inhibitor [112]. Alongside, chordin is involved in neural induction and mesoderm dorsalization during embryonic development. Deficiency of chordin leads to abnormalities in the skull, cardiovascular defects, malfunction in cervical and thoracic vertebrae, and also the absence of parathyroid and thymus [113]. Similarly, Noggin plays an important role in bone formation, and neural tissue formation during embryogenesis. Lack of Noggin leads to abnormalities in the skeleton and is lethal [114]. Animal studies have revealed that chordin deficiency results in stillborn mice [115] while noggin deficiency results in fetal death [114].

## 5. Regulators of BMP-7

The secreted proteins Chordin-like (CHRDL1), Crossveinless-2 (BMPER/CV-2), Kielin/chordin-like protein (KCP/CRIM2), and connective tissue growth factor (CTGF) act both as agonists and antagonists, depending on the particular ligands they regulate and the presence or absence of other factors in cell-type-specific microenvironment they encounter.

CHL/Neuralin (CHRDL1): Chordin-like (CHL/CHL1, CHRDL1) is a secreted molecule with three cysteine-rich repeat (CR) modules and is known as neuralin in the mouse [116,117,118]. CHRDL1 enhances BMP-4 and BMP-7 signaling in several cell lines when expressed alone. However, it switches into a selective BMP-7 antagonist when it complexes with Twsg1 and plays a role in inhibition of injury repair and homeostasis of the mammalian kidney [109].

Crossveinless-2 (BMPER/CV-2): CV-2 also known as BMPER, has been identified in mice, and humans. BMPER contains an additional carboxy-terminal trypsin inhibitor-like cysteine-rich domain. BMPER is expressed in mice at sites that require elevated BMP signals, such as the posterior primitive streak and ventral tail bud. CV-2/BMPER binds BMP-2, -4, -6, -7, -9. BMPER enhances BMP signaling during gastrulation, neural crest specification, nephrogenesis, cardiovascular development, and axial skeletal formation. It blocks BMP-9 in the vascular endothelium whereas, CV-2/BMPER overexpression showed the activities that are consistent with functions in both enhancing and inhibiting BMP signaling [119,120,121,122,123,124].

Kielin/chordin-like protein (KCP/CRIM2): CRIM2 contains 18 CR motifs and a carboxy-terminal vWF type D domain. KCP binds BMP-7 and increases BMP-7 binding to BMPRIA/ALK-3. It also enhances Smad1 activation and further promotes BMP-responsive gene expression and signaling to attenuate renal interstitial fibrosis [125]. It has been noticed that KCP also binds activin A and TGF-b1, and blocks Smad2/3 activation and inhibits Smad2/3-mediated transcription [125]. Hence, KCP functions in opposite ways to regulate activin/TGF-b and BMP signals. In addition, Soofi et al. demonstrated that KCP attenuates acute and chronic renal injury [126].

Connective tissue growth factor (CTGF): CTGF binds BMP-2,-4,-7 via its CR domain. Disruption of *CTGF* gene in mice revealed its requirement for coordination of chondrogenesis and angiogenesis during skeletal development [127], which depends on the CTGF activity to modulate BMP signaling during chondrocyte differentiation [128,129].

BMP signaling is controlled by different types of regulators, including extracellular matrix proteins (ECM), I-smads, ubiquitin proteasome complex, corepressors and miRNA. Based on the availability of ligands, ECM controls BMP signaling whereas I-smads antagonize the steps involved in smad signaling. Similarly, ubiquitin proteasome controls different types of inhibitors and signal transducers involved. Corepressors regulate BMP signaling at the transcriptional level and miRNAs regulate at the translational level [7].

## 6. BMP-7 as an Anti-Inflammatory Agent in Atherosclerosis

Atherosclerosis is a serious cardiovascular condition that involves the constriction of the arterial wall leading to the development of myocardial infarction. Atherogenesis is regulated by cholesteryl ester (CE) accumulation, foam cell formation, smooth muscle cell migration, necrotic core formation, and increased calcification [66,130,131,132]. Moreover, the developed atherogenesis creates turbulence in blood flow leading to plaque rupture and thrombosis. Although these atherogenic factors are well-established, recent data suggests the involvement of modified LDL, extracellular components in the plaque activation and rupture [133]. Therefore, atherosclerosis was considered to be the product of lipoprotein accumulation, particularly LDL in the arterial wall [134,135].

Recently, it is speculated that atherosclerosis is a complex process that involves the participation of both immune systems, oxidative stress, various cell types, receptors, lipids, enzymes, signaling pathways, trace elements, and other products [136,137,138]. Inflammation and oxidative stress are considered to be major players in the progression of the disease [139,140,141,142]. Altered vessel wall structure and disturbed blood flow patterns include inflammation and varied stress levels in developed atherosclerosis [143]. Despite the abundance of research literature on the topic, the role of lipids, especially fatty acids and their oxidation products like peroxidized linoleic acid (HPODE), 4-hydroxynonenal (HNE), oxo-nonanoic acid (ONA), and their interaction with inflammatory molecules such as oxidized LDL, phospholipids, TNF-α, vascular cell adhesion molecule (VCAM1) in many of these processes are poorly understood.

**Figure 3:**
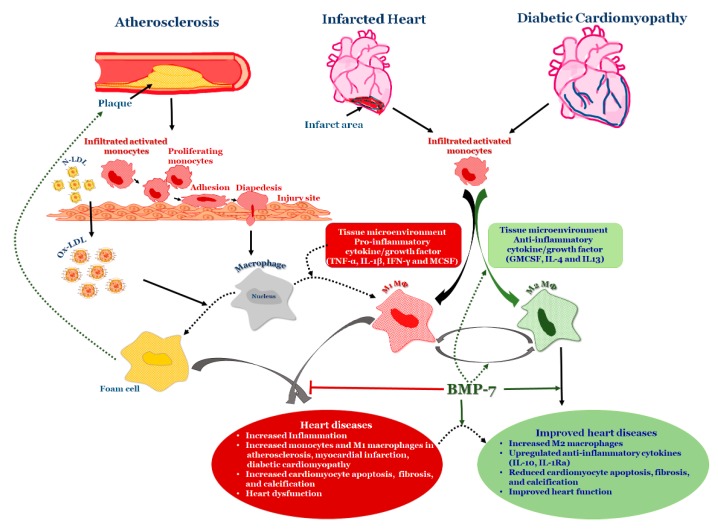
BMP-7 in Heart Diseases: Schematic representation of how BMP-7 modulates inflammation in heart diseases by converting infiltrated monocytes into anti-inflammatory M2 macrophages. LDL: Low-density lipoprotein; Ox-LDL: oxidized lipoprotein; TNF-α: Tumor necrosis factor alpha; IFN gamma: Interferon gamma; GMCSF: granulocyte macrophage colony stimulating factor; MCSF: macrophage colony stimulating factor.

Monocytes, which are precursors of macrophages as well as dendritic cells (DCs) and migrate into the areas of “injury” as a result of a chemotactic stimuli such as monocyte chemotactic protein 1 and 3 (MCP-1&3). Migration of monocytes into the arterial wall has been considered as one of the initial events in atherogenesis which persists in different stages of disease progression [140,141,142]. In tissues, based on the environmental growth factors and pro-inflammatory cytokines, monocytes differentiate into either M1 macrophages or DCs. Monocyte adherence, their differentiation into pro-inflammatory macrophages/dendritic cells that release pro-inflammatory cytokines which are involved in the generation of complex pathophysiology of atherosclerosis [140] (Figure 3). Macrophages were initially viewed as a mere scavenger of altered lipoproteins. However, the presence of macrophages along with lymphocytes in atherosclerotic plaques showed enhanced inflammatory immune response and release of pro-inflammatory molecules. The specific roles of different stages of atherosclerosis and presence of these inflammatory macrophages, foam cells, lymphocytes, and vascular smooth muscle cells are not yet completely understood. For example, M2 macrophages, are known for high endocytic clearance capacity due to their higher expression of scavenger receptors (SR) during wound healing and repair processes [144]. Van Tits et al. demonstrated that M2 macrophages are susceptible in forming foam cells in presence of oxidized LDL and shift towards the M1 phenotype with enhanced secretion of the pro-inflammatory cytokines IL-6, IL-8 and MCP-1 [145]. Furthermore, this increased production of pro-inflammatory cytokines by polarized M1 macrophages from M2 macrophages which are residing in subendothelial space of the vessel wall might lead to the initiation of the inflammatory cascade that mediates disease progression [145]. Similarly, in human atherosclerotic lesions different macrophage phenotypes exist in different plaque locations. M2 (CD68^+^ CD206^+^) macrophages were located in plaque stable zones far from the lipid core, whereas M1 (CD68^+^ CCL2^+^) macrophages exhibited a distinct tissue localization pattern [146] suggesting that the tissue microenvironment decides the fate of macrophage polarization. Subsequent research studies confirmed this finding by demonstrating the presence of lipid droplets in CD68^+^ CD206^+^ macrophages in comparison with CD68^+^ CD206^−^ macrophages [147]. This discovery suggests that despite the anti-inflammatory nature of M2 macrophages they tend to form foam cells, a significant contributor of atherogenesis.

We demonstrated from our laboratory that rhBMP-7 is able to inhibit the atherosclerosis associated inflammation at both acute (Day-14) and mid-stage (Day-28) time points of atherosclerosis by promoting monocyte differentiation into the anti-inflammatory M2 phenotype via reducing phosphorylated kinases p-38 and JNK while increasing p-Smad and ERK pathways [69,71]. Additionally, a recent study from our laboratory demonstrated the significantly increased BMPR2 expression on monocytes following BMP-7 treatment, and further polarization into M2 macrophages [67]. BMP-7 treatment showed increased M2 macrophages [approximately 25% at Day-14 and 60% at Day-28] than M1 macrophages [15% at Day-14 and 30% at Day-28] leading to decrease in pro-inflammatory cytokines such as tumor necrosis factor alpha (TNF-α), IL-6 and MCP-1 associated with atherosclerotic lesion development and to an increase in anti-inflammatory cytokines such as IL-10 and IL-1ra levels. Further, BMP-7 improved blood flow in the artery after post ligation, reduced the inflammatory kinases, and completely slowed down disease progression (Figure 3). In addition, we also demonstrated that, upon macrophage depletion by liposomal clodronate, BMP-7 fails to significantly reduce plaque progression and inflammation suggesting the direct role of BMP-7 on macrophages [71]. The literature on BMP-7 in macrophage polarization is new and growing; however, there are certain unanswered questions such as whether BMP-7 can inhibit the formation of foam cells; and if BMP-7 can inhibit the conversion of M2 macrophage into foam cell formation in atherosclerosis?

## 7. BMP-7 as an Anti-Calcifying Agent

Calcification is an important step in atherosclerosis as a result of inflammation and is classified into two main types; (1) intimal and (2) medial calcification [148,149,150]. Intimal calcification occurs during the progression of atherosclerotic lesions, whereas medial calcification occurs in between the layers of smooth muscle cells. Existing reports suggest that vascular calcification is a cell mediated process in which vascular smooth muscle cells (VSMCs) and pericytes, differentiate and mineralize the vascular matrix through abnormal deposition of calcium phosphate [148,149,150,151]. Recently, Riad et al. demonstrated the role of lipid peroxidation derived dicarboxylic acid, azelaic acid in calcium sequestration and subsequent calcification [152]. Evidence suggests that BMP-2 plays a major role in vascular calcification by inhibiting VSMC proliferation through p21 cyclin dependent kinases inhibition and subsequent cell cycle arrest [153,154,155,156,157]. In addition, it also causes the loss of smooth muscle cell markers while promoting the osteoblastic gene expression markers including ALP, OPN etc. by stimulating the osteogenic transcription factor Msx2 and inducing apoptosis, which is a critical step in calcification initiation. In contrast to BMP-2, BMP-7 counteracts atherosclerotic calcification by increasing SMC proliferation via upregulating p21 cyclin dependent kinases and regulating skeletal remodeling and maintaining SMC phenotype [158,159]. Several factors, including reactive oxygen species (ROS), reactive nitrogen species (RNS), vitamin D, phosphate, azeloate and parathyroid hormone increase the calcification process [152,160,161]. Various studies showed that BMP-7 inhibits vascular calcification by preserving the SMC phenotype and the process towards osteoblastic phenotype [156,157,162,163]. Kang et al. demonstrated that rhBMP-7 inhibited vitamin D and phosphate induced vascular calcification in vivo (mice) and in vitro (human aortic smooth muscle cells) [164]. In this study, C57BL/6J mice were treated with high concentrations of vitamin D in the presence and absence of rhBMP-7 and calcification markers were analyzed by IHC and western blotting. Vitamin D significantly increased osteoblastic markers (OPN and OCN) and calcium staining of aortas and hearts; whereas, pre-treatment with rhBMP-7 completely abolished the Vit-D mediated effects on osteoblastic markers and calcium staining. Further, these studies also demonstrated the efficacy of BMP-7 in attenuation of beta-glycerophosphate promoted osteogenic markers and calcium staining in vascular smooth muscle cells. Therefore, these studies also suggested the potential beneficial role of BMP-7 in reducing CVD related to vascular calcification [164].

## 8. BMP-7 Inhibits Inflammation and Adverse Remodeling in the Infarcted Heart

Myocardial infarction (MI) (Figure 3) is a condition due to the formation of lesions in the arteries, resulting in reduced blood flow of nutrients and oxygen supply, which leads to myocardial injury. Cardiac myocyte cell loss in the infarcted region happens via apoptosis, pyroptosis and necrosis leading to end stage heart failure [130,165,166,167,168]. Furthermore, cardiac hypertrophy and fibrosis has been considered as a major remodeling mechanism to compensate for the requirements under pathophysiological conditions in which increased cardiac cell size (hypertrophy) and expression ofECM proteins (collagens types I and III) have been observed [169,170]. In the injured myocardium, fibrosis stiffens the heart muscle and affects the systolic and diastolic function. Moreover, these ECM proteins can be degraded by endopeptidases such as matrix metalloproteinases (MMPs) leading to alterations in ventricular structure and function post-MI. Moreover, following myocardial injury, the heart undergoes a sequence of molecular events including cell death, cytokine release, and infiltration/recruitment of immune cells, which play a major role in cardiac wound healing and stabilization of cardiac remodeling [171].

After 48–72 h of MI, monocytes were recruited to the infarct area of the heart in two phases [172]. In the first phase, a significant increase in number of Ly-6c^high^ monocytes are observed in the MI which are chemokine receptor type 2 (CCR2) dependent [173,174]. These monocytes secrete TNF-α and IL-1β and are converted to pro-inflammatory macrophages to clear the debris of dead cells and extracellular matrix by phagocytosis. It is postulated that monocytes infiltrated in response to cardiac cellular injury to clear the dead cardiac cells, and also generate an inflammatory microenvironment, that triggers adverse cardiac remodeling. In the second phase, Ly-6c^low^ monocytes are recruited which are C-X3-C motif chemokine receptor (CX3CR1) dependent [175]. These monocytes are less in number, but convert to macrophages, which play a role in wound healing and repair [176] by promoting collagen deposition, angiogenesis, and myofibroblast accumulation. These infiltrated monocytes interact with ECM and release fibronectin, which stabilizes/reduces the infarct [176]. In addition, early efferocytosis promotes the conversion of M1 macrophages into M2 macrophages, reduces secretion of pro-inflammatory cytokines and increases production of anti-inflammatory cytokines IL-10 and TGF-β [177,178,179].

Cell death due to apoptosis has been considered as a key step in the development and progression of post-MI remodeling which further leads to chronic heart failure [180,181]. Apoptosis is a type of programmed cell death, which occurs during aging and development as a homeostatic process as well as a defense mechanism in various diseases. Apoptosis leads to a cascade of cellular events including cytoplasmic blebbing, cell shrinkage, protein cleavage by caspases, chromatin condensation and DNA fragmentation [182]. Cardiac myocyte apoptosis is mediated through extrinsic and intrinsic pathways. TNF-α, FAS ligand, and TNF-related apoptosis-inducing ligand (TRAIL) triggers the extrinsic pathway whereas caspases triggers the intrinsic pathway [183]. Further, cardiac myocyte apoptosis provides a microenvironment to infiltrate monocytes, and initiates inflammation that activates cardiac fibroblasts, which play a major role in the cascade of inflammation, cellular infiltration, and fibrosis in both infarcted and peri-infarcted areas. These cellular alterations lead to adverse cardiac remodeling that generates organ dysfunction. Interstitial fibrosis occurs between cardiac myocytes whereas vascular fibrosis occurs in and around vessel walls [182,184]. Several pro-inflammatory as well as profibrotic cytokines released by leukocyte infiltration lead to fibroblasts activation, increased TGF-β secretion and ECM protein synthesis [185,186,187,188,189,190,191,192].

The role of TGF-β1 in inflammation and cardiac injury is reported in myocardial infarction [5,193,194]. Upregulation of TGF-β activates Smad signaling proteins 2,3,4 in the infarct area of the heart and also the peri-ischemic zone under pathological conditions [193,195], which might play a role in increased collagen type-I expression [196]. Schneiders et al. demonstrated the involvement of Smad proteins in cardiomyocyte apoptosis [197]. It has been noticed that cardiomyocyte treatment with TGF-β1 enhances cardiomyocytes apoptosis, increases caspase3/7 activity and decreases B-cell lymphoma 2 (Bcl-2) expression by upregulating Smad-7 [198]. Activation of the TGF-β/Smad pathway leads to increased ECM components, such as fibronectin, type-1 collagen, connective tissue growth factor (CTGF), and transcription genes related to the collagen production, which leads to fibrosis development [199]. It has been reported that overexpression of TGF-β1 in mice showed a significant increase in left ventricular fibrosis [200]. In addition, TGF-β1 is known to increase plasminogen activator inhibitor-1 which plays a major role in ECM degradation [200,201]. Evidence suggests that cardiac fibrosis is mediated by TGF-β/Smad signaling [202,203,204]. Smad-4 plays a role in initiation of Smad-2/3 associated TGF-β induced fibrosis whereas Smad-7 inhibits collagen, smooth muscle actin, and reduces matrix protein by inhibiting phosphorylation of Smad-2/3 [205].

BMP-7 acts as an antifibrotic factor through the Smad pathway in which it induces the phosphorylation of Smad-1/5/8 and downregulates TGF-β signaling, which is mediated by Smad-2/3 phosphorylation [206]. The downregulation of BMP-7 in pathological fibrosis of organs has been reported [207,208,209,210,211,212]. Additionally, administration of exogenous BMP-7 or overexpression of BMP-7 protects the tissues such as kidneys [207,210], liver [208], lungs [209] and heart [211] from fibrosis. Moreover, exogenous administration of BMP-7 downregulates myocardial interstitial fibrosis as well as kidney fibrosis by inhibiting TGF-β signaling pathway and protects cardiac function. Recently, Jin et al. demonstrated that exogenous BMP-7 facilitates cardiac function recovery after acute myocardial infarction by attenuating myocardial fibrosis through counteracting TGF-β1 signaling pathway [211]. In this study, the group established acute myocardial infarction by ligating the left anterior descending artery with and without BMP-7 treatment. BMP-7 treatment significantly attenuated myocardial fibrosis, reduced the infarct size, and improved cardiac function. In addition, this study also reported that BMP-7 treatment inhibited myocardial fibrosis by attenuating TGF-β signaling and its downstream effectors Smad-2 and Smad-3 [211]. Furthermore, the beneficial role of BMP-7/Smad signaling has been shown in fibrotic disease of the heart [212,213]. However, the direct role of BMP-7 in monocyte differentiation or M1 macrophage polarization into M2 macrophages in the infarcted heart is still unknown.

## 9. BMP-7 Ameliorates Diabetic Cardiomyopathy

Diabetes is a major metabolic disorder and an alarming epidemic affecting millions of people globally. It is considered as the seventh leading cause of death [214]. Pre-diabetes is a condition in which impaired glucose levels have been considered as markers for diagnosis which leads to type 1 and type 2 diabetes (T2DM) [215]. Type I diabetes is known as an autoimmune disease caused by insulin deficiency due to the destruction of insulin producing pancreatic beta cells of the islets of Langerhans [216]. Similarly, T2DM is characterized by insulin resistance resulting from impairment of the normal function of pancreatic β-cells which induces hyperglycemia, and eventually leads to cardiac failure and nephropathy [217,218,219]. Diabetes is usually accompanied with hyperglycemia, oxidative stress, and inflammation potentially leading to CVD, muscle atrophy, nephropathy, neuropathy, periodontal disease, retinopathy, impaired wound healing, and tissue damage [217,218,219].

Diabetic cardiomyopathy (DC) is the leading cause of death worldwide and has attracted global attention. Due to the fact that low levels of glucose can initiate microvascular complications; therefore, the impaired glucose tolerance has been considered as a major risk factor for CVD related deaths [220]. Increased inflammation and oxidative stress have been observed in both clinical and experimental diabetes mellitus which are also implicated in the etiology of chronic diabetic complications [221,222,223,224] such as diabetes-induced cardiomyopathy and muscle toxicity. However, the exact mechanisms of developed chronic diabetic complications are not yet completely understood. Diabetes results from the functional imbalance of innate and adaptive immune response [225]. The elevated blood levels of pro-inflammatory cytokines such as TNF-α and IL-6 have been noticed in diabetic subjects [226,227]. Recent studies have suggested that increased levels of IL-6 should be considered as a risk factor for diabetes [228]. Inhibition and decreased expression of TNF-α and IL-6 might play a possible role in alleviating diabetic complications.

TNF-α is known to induce cardiomyocyte apoptosis in vitro by initiating the apoptotic cascade via caspase-3 triggering in vitro [229]. Cardiac apoptosis can be induced by various mechanisms, including oxidative stress, inflammatory cytokines, loss of normal insulin signaling, hyperglycemia and advanced glycation end products (AGEs). Recent evidence suggested the upregulation of cardiomyocyte apoptosis in diabetic subjects as well as in animal models [230,231,232,233].

Izhumi et al. demonstrated the ability of BMPs in attenuating apoptosis in rat cardiomyocytes. According to this study, BMP-2 can attenuate the serum deprivation induced apoptosis in cardiac myocytes. In addition, these studies elucidated the up-regulation of B-cell lymphoma-extra-large (Bcl-xL) via the Smad-1 pathway, which has a protective effect and plays an important role in regulation of the myocardium [233]. Studies from our laboratory correlated with these in vitro studies in that BMP-7 attenuates cardiac myocyte apoptosis in diabetes-induced mice [234]. BMP-7 treated pre-diabetic group has shown significantly increased levels of anti-inflammatory IL-10 and reduction in TNF-α [234]. IL-10 has been known to decrease TNF-α induced cardiomyocyte apoptosis [72]. Further, BMP-7 reduced diabetic cardiac apoptosis is mediated through Phosphatase and tensin homolog (PTEN) and Akt pathways [234]. Elevated levels of PTEN protein were observed in pre-diabetic mice hearts as compared to control mice whereas significant downregulation of PTEN was observed in BMP-7 treated pre-diabetic mice. In addition to anti-apoptotic effects of BMP-7, this study also reported the anti-fibrotic effects that leads to improved cardiac function in pre-diabetic mice [234]. According to Kurlawalla et al., PTEN decreases insulin sensitivity, and lack of PTEN increases glucose tolerance and insulin sensitivity in adipose tissue. It has been shown that PTEN knock-out mice are resistant to streptozotocin (STZ)-induced diabetes which might suggest PTEN as promising target to aim in reversing insulin resistance [235]. Moreover, BMP-7 inhibits apoptosis via PTEN-Akt pathway and decreases hyperglycemia in pre-diabetic mice.

## 10. BMP-7 Differentiates Monocytes into M2 Macrophages in Heart Diseases

Monocyte polarization plays a key role in the progression of various inflammatory diseases such as atherosclerosis, myocardial infarction, and diabetic cardiomyopathy [236,237,238]. Infiltrated monocyte differentiation depends on the tissue microenvironment they reside in and the external stimuli they receive [239]. Monocytes will differentiate into M1 macrophages if tissues have an inflammatory microenvironment stimulated with interferon gamma (IFN-γ), macrophage colony stimulating factor (MCSF) and TNF-α [240]. Conversely, infiltrated monocytes will polarize into M2 macrophages or alternative macrophages if the tissue microenvironment is surrounded by certain specific factors, such as granulocyte macrophage colony stimulating factor (GMCSF) and anti-inflammatory cytokines, such as IL-4 and IL-13 [187]. In addition, two distinct subsets of M2 macrophages, M2a and M2c are notable in which the former participates in wound healing and are induced by IL-4 and IL-13 whereas the latter takes part in regulation of disease progression, which is induced by glucocorticoids, TGF-β and IL-10 [187,241]. Differentiated M1 macrophages are known to secrete pro-inflammatory cytokines such as inducible nitric oxide synthase (iNOS), IL-6, TNF-α, and MCP-1, while alternative M2 macrophages are known to secrete anti-inflammatory cytokines as such IL-10 and arginase-1 [67,242,243,244].

The exact role of infiltrated monocytes and their differentiation into pro-inflammatory M1 and anti-inflammatory M2 macrophages, as well as their role in development and progression at different stages of the diseases of atherosclerosis, myocardial infarction, and diabetic cardiomyopathy is far from clear; however, we are beginning to understand that increased M2 macrophages attenuate developed cardiac pathophysiology and function. In early onset of disease, monocytes move to the injury site and polarize into M2 macrophages [65] to repair by secreting anti-inflammatory cytokines such as IL-10 and IL-1Ra as well as scavenge the apoptotic cells [245,246,247]. As the disease progresses, the infiltrated monocytes polarize into M1 macrophages due to the microenvironment resulting in increased secretion of pro-inflammatory cytokines including MCP-1, TNF-α and IL-6, further increasing the necrotic core formation and calcification [223,248]. Balancing the ratio of M1 to M2 could control the severity of disease progression.

Considering the beneficial effects of M2 macrophages in the attenuation of inflammation, wound healing, and repair processes, the factors/molecules/compounds that have the ability to convert the monocytes into M2 macrophages have attained major attention due to their potential therapeutic implications. BMP-7 is one such factor which has the ability to polarize monocytes into M2 macrophages in both normal as well as under stressed conditions. A recent study demonstrated the potential efficacy of BMP-7 in monocyte polarization to M2 macrophages by upregulating M2 macrophage marker CD206, and down regulating the monocyte marker CD14 [67]. In addition, it was also noticed that BMP-7 significantly reduced pro-inflammatory cytokines such as IL-6, TNF-α, MCP-1, but enhanced the anti-inflammatory cytokines secretion of IL-10 and IL-1Ra [67] suggests that BMP-7 has a potential to enhance M2 macrophages which are anti-inflammatory in nature. Further, this study suggests that M2 macrophage polarization decreases the activation of the inflammatory p38 and JNK pathways while increases the activation of Smad and ERK pathways at mid-stage (Day-28) time point of atherosclerosis [69].

BMP-7 upregulates and binds BMPR2, phosphorylates SMAD1/5/8, and activates PI3K, which results in downstream activation of Akt and mTOR as shown in Figure 2. Evidence demonstrated that the expression of p-PTEN, an inhibitor of the PI3K pathway was significantly upregulated in apoptotic conditions and significantly downregulated upon BMP-7 treatment, suggesting the ability of BMP-7 to not only promote PI3K signaling through upregulated mediators but also by directly blocking inhibition of the signaling cascade [70,97]. Furthermore, these studies also demonstrated how BMP-7 inhibitor follistatin inhibitied p-SMAD1/5/8 expression and decreased PI3K expression, which supports and suggests the necessity of BMP-7 to bind BMPR2 thus activating SMAD1/5/8 and subsequently PI3K [111,249]. As mentioned above, the PI3K pathway plays a key role in increased anti-inflammatory markers such as arginase-1 and IL-10 as well as inhibition of the production of pro-inflammatory markers [97,250,251]. It has been noticed that activation of the PI3K pathway resulted in increased polarization of M2 macrophages, specifically in bone marrow derived macrophages [98]. Evidence has also suggested that inhibition of either PI3K or mTOR results in M1 macrophage polarization signifying the role of these pathways in monocyte polarization into M1 macrophages [98,250]. Moreover, Rocher et al. demonstrated that BMP-7 administration along with apoptotic conditional medium to monocytes resulted in an increased expression of anti-inflammatory cytokines (IL-1ra, IL-10 and arginase-1) and inhibited expression of pro-inflammatory cytokines (iNOS, TNF-alpha, IL-6 and MCP-1) which promoted paracrine effects on monocytes and macrophages yielding increased M2 macrophage polarization [70]. According to Mantovani et al. M2 macrophages counteract inflammation by enhanced secretion of IL-10 [252]. It has also been reported that these anti-inflammatory cytokines have the ability to inhibit pro-inflammatory cytokines IL-6 and TNF-α from immune cells and can be used as therapeutic agents [253] in several inflammation-associated diseases.

## 11. Conclusions and Future Directions

In conclusion, these studies support that BMP-7 is an effective growth factor that has the potential to inhibit apoptosis, fibrosis and acts as an anti-calcifying agent, which ultimately improves cardiac function in different heart diseases, as summarized in this review. The novel and most interesting role of BMP-7 is its ability to promote the differentiation of infiltrated pro-inflammatory monocytes into anti-inflammatory M2 macrophages in different cardiac diseases. However, further studies are required to understand whether BMP-7 can acts as a direct anti-inflammatory agent to inhibit cardiac pathophysiology. Further investigation is needed to determine if BMP-7 treatment differentiates monocytes or polarizes M1 to M2 macrophages, and whether it can be reverted due to low concentration of BMP-7. It is also not yet clear whether a single dose of BMP-7 is enough to decrease diabetes and diabetic cardiomyopathy as long-term studies are not well established. Therefore, a new significant research avenue remains to be explored to understand the cell protective role of BMP-7 in treating heart diseases.

## Figures and Tables

**Table 1:** Types of bone morphogenetic proteins (BMPs) and their functions.

Types	Alternate Names	Tissues that Express	Functions	Receptors
BMP-1	BMP-1 is a metalloproteinase	major end organs (heart, lung, liver, pancreas, kidney, and brain), lymphoid organs (bone marrow, thymus, spleen and lymph nodes), exocrine glands (prostate and mammary gland) organ protectors (muscle and bone)	Metalloprotease that cleaves COOH–propeptides of procollagens I, II, and III/induces cartilage formation/cleaves BMP antagonist chordin	_____
BMP-2	BMP-2A, XBMP2, xBMP-2, MGC114605	major end organs (lung, pancreas, and kidney), lymphoid organ (spleen)	Induces bone and cartilage formation. Plays a role in skeletal repair and regeneration/heart formation	ALK-2, 3, 6 BMPR-II; ActR-IIA, ActR-IIB
BMP-3a & 3b	Osteogenin, BMP-3A	major end organs (brain, heart, pancreas), exocrine gland (prostate), organ protector (skeletal muscle), lymphoid organs (bone marrow, spleen and thymus), BMP-3b also expresses in spinal cord	Negative regulator of bone morphogenesis Cell differentiation regulation; skeletal morphogenesis; Regulates cell growth and differentiation in both embryonic and adult tissues	ALK-4 ActR-IIA, ActR-IIB
BMP-4	BMP-2B, BMP2B1, ZYME, OFC11, MCOPS6	major end organs (brain, heart, pancreas, liver, lung, kidney), exocrine gland (prostate), organ protector (skeletal muscle), lymphoid organs (bone marrow, spleen and thymus), spinal cord	Skeletal repair and regeneration; kidney formation; Induces cartilage and bone formation; limb formation; tooth development.	ALK-2,3,5,6 BMPR-II, ActR-IIA
BMP-5	MGC34244	major end organs (brain, heart, pancreas, liver, lung, kidney), exocrine gland (prostate), organ protector (skeletal muscle), lymphoid organs (bone marrow, spleen and thymus), spinal cord	Limb development; induces bone and cartilage morphogenesis; connecting soft tissues	ALK-3 BMPR-II; ActR-IIA, ActR-IIB
BMP-6	Vgr1, DVR-6	major end organs (brain, heart, pancreas, liver, lung, kidney); exocrine gland (prostate); organ protector (muscle and bone), lymphoid organs (bone marrow, spleen and thymus); spinal cord	Cartilage hypertrophy; bone morphogenesis; nervous system development; Plays a role in early development	ALK-2, 3, 6 BMPR-II; ActR-IIA, ActR-IIB
BMP-7	OP-1	major end organs (brain, heart, pancreas, liver, lung, kidney), exocrine gland (prostate) organ protector (skeletal muscle), lymphoid organs (bone marrow, spleen and thymus), spinal cord.	Skeletal repair and regeneration; kidney and eye formation; nervous system development plays a major role in calcium regulation and bone homeostasis	ALK 2, 3, 6 BMPR-II;
BMP-8a & 8b	OP-2, FLJ14351, FLJ45264 OP-3, PC-8, MGC131757	major end organs (brain, heart, kidney, lung, liver, pancreas), exocrine gland (prostate), organ protector (skeletal muscle), lymphoid organs (spleen, thymus bone marrow) spinal cord	Induces cartilage formation; Bone morphogenesis and spermatogenesis; calcium regulation and bone homeostasis.	ALK 2; 3; 4; 6; 7 BMPR-II; ALK3,6 BMPR-II; ActR-IIA, ActR-IIB
BMP-9	GDF-2	major end organ (liver)	Bone morphogenesis; cholinergic neurons development; in glucose metabolism; potent inhibitor of angiogenesis	ALK-1,2 BMPR-II; ActR-IIA, ActR-IIB
BMP-10	MGC126783	major end organs (brain, heart, kidney, lung, liver, pancreas), exocrine gland (prostate), organ protector (skeletal muscle), lymphoid organs (spleen, thymus, bone marrow) spinal cord.	Heart morphogenesis maintains the proliferative activity of embryonic cardiomyocytes by preventing premature activation of the negative cell cycle regulator; inhibits endothelial cell migration and growth	ALK-1, 3, 6 ActR-IIA, ActR-IIB
BMP-11	GDF-11	major end organs (brain, pancreas), exocrine gland (prostate), lymphoid organs (spleen, thymus bone marrow) spinal cord.	Pattering mesodermal and neural tissues, dentin formation	ALK-3, 4, 5, 7 BMPR-II; ActR-IIA, ActR-IIB
BMP-12	GDF-7, CDMP-3	_____	Ligament and tendon development/sensory neuron development	ALK-3, 6 BMPR-II; ActR-IIA
BMP-13	GDF-6, CDMP-2, KFS, KFSL, SGM1, MGC158100, MGC158101	_____	Normal formation of bones and joins; skeletal morphogenesis and chondrogenesis Plays a key role in establishing boundaries between skeletal elements during development	ALK-3, 6 BMPR-II; ActR-IIA, ActR-IIB
BMP-14	GDF-5, CDMP-1, OS5, LAP4, SYNS2, MP52	sensory organs (eye, skin), major end organs (brain, heart; kidney, liver, lung), embryonic tissue, mixed connective tissue, pituitary gland, salivary gland; exocrine gland (prostate), reproductive system related (uterus), lymphoid organ (bone marrow)	Bone and cartilage formation; Skeletal repair and regeneration	ALK-3, 6 BMPR-II; ActR-IIA
BMP-15	GDF-9B, ODG2, POF4	_______	Oocyte and follicular development	ALK-6
BMP-16	_____	embryonic tissue; reproductive system (testis)	Skeletal repair and regeneration Essential for mesoderm formation and axial patterning during embryonic development	_____
BMP-17	_____	major end organ (brain, lung, liver, pancreas, spleen) lymphoid organ (lymph node); exocrine gland (mammary gland); sensory organ (skin); reproductive organ (testis); bladder; embryonic tissue; intestine; joints;	Required for left-right axis determination as a regulator of LEFTY2 and NODAL	_____
BMP-18	_____	major end organ (brain), embryonic tissue, reproductive system (testis)	Required for left-right (L-R) asymmetry determination of organ systems in mammals. May play a role in endometrial bleeding	_____

ALK: activin receptor-like kinase; Actr: activin receptor; BMPR: bone morphogenetic protein receptor.

**Table 2:** Types of antagonists and their functions.

Inhibitors	Name	Role
Neuroblastoma Dan family	DAN	plays a role in tumor suppression; cell proliferation
	PRDC/GRem2 (protein related to DAN and Cerberus)	PRDC is a secreted, cysteine knot-containing BMP antagonist; play a role in regulation of BMP signaling in ovary, brain, and other adult tissues
	Gremlin	higher level expression in Basal cell carcinoma stromal cells; promotes proliferation and tumor growth; induces cell cycle progression via p21; interacts directly with target endothelial cells; acts as a proangiogenic factor to regulate angiogenesis; blocks osteoblast differentiation and function by blocking BMP signaling
	Cerberus/Cer1	anterior neural induction and somite formation during embryogenesis; regulate Nodal signaling during gastrulation as well as the formation and patterning of the primitive streak Blocks Nodal, BMP, and Wnt signaling
	Coco/Dand5	antagonizes NODAL and BMP4 signaling during development, organogenesis, tissue growth and differentiation; Blocks BMP/TGF-β and Wnt signaling
	Caronte	antagonizing symmetrically expressed BMP signals
	USAG-1	BMP and Wnt antagonist during the development of kidney, tooth, and mammary tissues
	Sclerostin/ SOST	endogenous antagonist of the Wnt/β-catenin pathway in the regulation of bone mass; acts as negative regulator of bone formation
	Dante/Dte	plays potential role during early stages of mouse embryonic development; inhibit BMP signaling
Chordin family	Chordin,	functions as BMP antagonist that blocks BMP activity by binding to the BMPs and inhibiting their interaction with their receptors
	Ventroptin/ Chordin-like-1/ Neuralin 1	regulates retinal angiogenesis via modulation of BMP4 actions in endothelial cells.
	Chordin-like-2,	prevents the binding of BMPs to type 1 and type 2 receptors as well as BMP-induced cellular responses; reduces the rate of matrix deposition by mesenchymal cells, acts as a negative regulator of cartilage formation.
	Kielin	enhances BMP signaling in a paracrine way; inhibits both the activin-A and TGFB1-mediated signaling pathways
	Nell	promotes the osteogenic differentiation of adipose-derived stromal/stem cells and inhibits adipogenic differentiation. Binding of NELL1 to Integrin beta 1 was shown to be critical for its role in promoting osteogenic differentiation and adhesion to the extracellular matrix.
	Crossveinless2	bone morphogenetic protein-binding endothelial cell precursor-derived regulator (BMPER). Secreted CV-2 interacts with BMP and inhibits its function
	Brorin	Brorin binds and antagonizes BMPs, interacting via the von Willebrand factor C domain. It promotes neurogenesis in mouse neural precursors
	Noggin	promotes skin tumorigenesis; reduces tumor size and decreases bone loss compared to untreated control animals; suppresses BMP4 induction of vascular endothelial growth factor receptor 2 in embryonic blood vessels; inhibits BMP interaction with their receptors
	Follistatin	acts as a modulator of gonadal tumor progression and the activin stimulated wasting syndrome; inhibits BMP interaction with their receptors
Twisted gastrulation		regulates the extracellular availability of a mesoderm inducer, BMP 4 As agonist-enhances cleavage of BMP/chordin complex by BMP1/tolloid (releasing free BMP) Required to specify the dorsal-most structures in embryo.
Follistatin-related gene (FLRG)		acts as activin antagonist and inhibits tumor cell growth

Note: Information obtained from Ref 3, 89-94 and some information from Uniprot. DAN: differential screening-selected gene aberrant in neuroblastoma; USAG1: uterine sensitization-associated gene1.

## Abbreviations

ALP	alkaline phosphatase
Akt	RAC-alpha serine/threonine-protein kinase
ATF2	activating transcription factor 2
Bcl-2	B-cell lymphoma 2
BMP	bone morphogenetic protein
BMPR	bone morphogenetic protein receptor
BRAM1	bone morphogenetic protein receptor associated molecule 1
CCR2	C-C chemokine receptor type 2
CE	cholesteryl ester
CTGF	connective tissue growth factor
CVD	cardiovascular disease
CX3CR1	C-X3-C motif chemokine receptor
DC	dendritic cells
ECM	extracellular matrix
ERK	extracellular signal-regulated Kinase
GMCSF	granulocyte macrophage colony stimulating factor
HSC	hematopoietic stem cell
IFN-γ	interferon gamma
iNOS	inducible nitric oxide synthase
JNK	c-Jun-N terminal Kinase
MAPK	mitogen-activated protein kinases
MCP-1	monocyte chemotactic protein
MCSF	macrophage colony stimulating factor
MMP	matrix metalloproteinase
MNC	mononuclear cells
MSC	mesenchymal stem cells
mTOR	mammalian target of rapamycin
MyoD	myoblast determination protein 1
NFkB	nuclear factor kappa-light-chain-enhancer of activated B
OCN	osteocalcin
OPN	osteopontin
OSX	osterix
PI3	phosphatidylinositol 3
PK	protein kinase
PTEN	phosphatase and tensin homolog
RON	reactive nitrogen species
ROS	reactive oxygen species
Runx2	run-related transcription factor 2
SR	scavenger receptor
T2DM	type 2 diabetes
TAB1	TAK1 binding protein
TAK1	TGF-beta activated kinase 1
TF	tissue factor
TGF-β1	transforming growth factor beta1
TNF-α	tumor necrosis factor alpha
TRAIL	TNF-related apoptosis-inducing ligand
VSMC	Vascular smooth muscle cells
XIAP	X-linked inhibitor of apoptosis protein
